# Diversity and distribution of Epeorus (Caucasiron) (Ephemeroptera, Heptageniidae) in Iran, with descriptions of three new species

**DOI:** 10.3897/zookeys.947.51259

**Published:** 2020-07-08

**Authors:** Ľuboš Hrivniak, Pavel Sroka, Jindřiška Bojková, Roman J. Godunko, Javid Imanpour Namin, Samereh Bagheri, Farshad Nejat, Ashgar Abdoli, Arnold H. Staniczek

**Affiliations:** 1 Biology Centre of the Czech Academy of Sciences, Institute of Entomology, Branišovská 31, 37005 České Budějovice, Czech Republic Institute of Entomology České Budějovice Czech Republic; 2 Faculty of Sciences, University of South Bohemia, Branišovská 31, 37005 České Budějovice, Czech Republic University of South Bohemia České Budějovice Czech Republic; 3 Department of Botany and Zoology, Masaryk University, Kotlářská 2, 61137 Brno, Czech Republic Masaryk University Brno Czech Republic; 4 Department of Invertebrate Zoology and Hydrobiology, University of Łódź, Banacha 12/16, 90237 Łódź, Poland University of Łódź Łódź Poland; 5 Department of Fishery, Faculty of Natural Resources, University of Guilan, POB 1144, Sowmehsara-Rasht, Iran University of Guilan Sowmehsara-Rasht Iran; 6 Department of Biodiversity and Ecosystem Management, Environmental Sciences Research Institute, Shahid Beheshti University, Daneshjou Boulevard, 1983969411 Tehran, Iran Shahid Beheshti University Tehran Iran; 7 Department of Entomology, State Museum of Natural History Stuttgart, Rosenstein 1, 70191 Stuttgart, Germany State Museum of Natural History Stuttgart Stuttgart Germany

**Keywords:** barcoding, Caucasus, diversity, mayflies, Middle East, taxonomy

## Abstract

Combining morphological and molecular data in an integrative approach, three new mayfly species of Epeorus (Caucasiron) are described. These include Epeorus (Caucasiron) alborzicus Hrivniak & Sroka, **sp. nov.** and Epeorus (Caucasiron) shargi Hrivniak & Sroka, **sp. nov.** from northern Iran, and Epeorus (Caucasiron) zagrosicus Hrivniak & Sroka, **sp. nov.** from central Iran. They are unambiguously delimited using both distance-based and likelihood-based approaches in the analyses of barcode COI sequences. Each new species is compared with other species of the subgenus and morphological diagnostic characters are provided. Based on extensive sampling of streams throughout the country, the distribution and habitat preferences of all *Caucasiron* species in Iran are assessed. Altogether, there are now six species recorded, among them also E. (C.) nigripilosus Sinitshenkova, 1976 is reported for the first time in Iran. Five species are distributed in the Alborz Mts. in northern Iran, one species was found in the Zagros Mts. in central Iran.

## Introduction

The genus Epeorus Eaton, 1881, subgenus Caucasiron Kluge, 1997 represents a group of mountainous mayflies distributed in Palaearctic region. Kluge (1997) defined *Caucasiron* based on a unique larval apomorphy, a projection on the costal margin of gill plates II–VII. Other larval diagnostic characters include the presence of medio-dorsally directed hair-like setae along anterior margin of head and gill plates forming a so-called "adhesive disc", consisting of enlarged gill plate I and overlapping gill plates II–VII. Gill plate VII has a longitudinal fold allowing to bend the plate ventrally under the abdominal segments. The systematic position of *Caucasiron* within *Epeorus*-related taxa was unclear for a long time (e.g., Braasch 2006, Kluge 2015). The recent study by Hrivniak et al. (2020) confirmed its monophyly and subgeneric position within *Epeorus* s.l. Moreover, the study pointed out its close phylogenetic relationship with the subgenusIron Eaton, 1883 distributed in Central Asia and Nearctic realm.

*Caucasiron* occurs in the Eastern Mediterranean (Samos and Cyprus Island), Anatolia, Caucasus, and central and western Asia (Hrivniak et al. 2019, 2020). Their larvae inhabit riffle sections of montane and submontane streams with coarse bed substrate (Nguyen et al. 2004; Bauernfeind and Soldán 2012). At present there are 17 species described (Hrivniak et al. 2020), but apparently several Central Asian taxa described in the genus *Iron* rather belong to *Caucasiron* (Chen et al. 2010; Hrivniak et al. 2017). In any case, a taxonomic revision of these species is needed to clarify their systematic position.

The highest species richness of *Caucasiron* and a remarkable regional and local endemism was found in the Caucasus Mountains (Hrivniak et al. 2017; Hrivniak et al. 2020), which represent one of the world biodiversity hotspots (Myers et al. 2000). The 12 species known from the Caucasus and adjacent areas are as follows: E. (C.) caucasicus (Tshernova, 1938), E. (C.) znojkoi (Tshernova, 1938), E. (C.) nigripilosus (Sinitshenkova, 1976), E. (C.) magnus (Braasch, 1978), E. (C.) alpestris (Braasch, 1979), E. (C.) soldani (Braasch, 1979), E. (C.) sinitshenkovae (Braasch & Zimmermann, 1979), E. (C.) longimaculatus (Braasch, 1980), E. (C.) bicolliculatus Hrivniak, 2017, E. (C.) turcicus Hrivniak, Türkmen & Kazancı, 2019, E. (C.) iranicus (Braasch & Soldán, 1979), and E. (C.) insularis (Braasch, 1983). The latter two species for a long time were considered as subspecies of E. (C.) caucasicus and E. (C.) znojkoi, respectively. The recent molecular study of the Caucasian *Caucasiron* fauna, however, confirmed all morphologically defined species/subspecies as distinct evolutionary lineages and, consequently, both subspecies were raised to species level (Hrivniak et al. 2020). Moreover, the delimitation of several additional evolutionary lineages indicated that the diversity of *Caucasiron* in the Caucasus region could be even higher. However, these lineages have remained without formal description to date (Hrivniak et al. 2020).

Individual *Caucasiron* species exhibit different distribution patterns within the Caucasus region varying from an endemic distribution in the Greater Caucasus to a wide distribution covering distant regions in the Pontic Mountains, Lesser Caucasus, Zagros, and Alborz Mountains (Hrivniak et al. 2020). The highest species richness and endemism of *Caucasiron* is concentrated in the western and central part of the Greater Caucasus, the most prominent mountain range in the Caucasus region. However, the individual mountain ranges of the Caucasus have been studied to a different extent until now. Especially the Alborz Mountains, a southeast part of the Caucasus biodiversity hotspot, and the Zagros Mountains, a dominant part of the Irano-Anatolian biodiversity hotspot, have been left unattended without detailed investigation (Bojková et al. 2018). The only *Caucasiron* species described and known exclusively from Iran, E. (C.) iranicus (Braasch & Soldán, 1979), is distributed in the Alborz and most likely represents an endemic species of this mountain range. However, given the size and diversity of the Iranian territory and stream habitats, the diversity and endemism within *Caucasiron* can be expected to be much higher in Iran. Summarizing recent knowledge on the diversity and distribution of Iranian mayflies, Bojková et al. (2018) reported two species of *Caucasiron* from Iran, namely E. (C.) iranicus and E. (C.) znojkoi.

Based on morphology and molecular analyses, we describe in this integrative study, two new species of *Caucasiron* from the Alborz Mountains and one new species from the Zagros Mountains. We provide morphological diagnostic characters of the three new species and differential diagnoses between all species known from the Caucasus and adjacent areas, plus an analysis of respective COI sequences. Following recent studies on Iranian mayflies by Bojková et al. (2018), Sroka et al. (2019), and Staniczek et al. (2020), we also sum up all records of *Caucasiron* species from our recent Iranian field trips to further contribute to a systematic research of mayflies in Iran.

The main objectives of this study are to (i) describe the morphology of three new *Caucasiron* species and provide their differential diagnoses, (ii) apply the molecular species delimitation methods using analytical tools for the single-locus COI dataset, (iii) provide basic information about habitat requirements of the new species, and (iv) summarize the distribution of all *Caucasiron* species recently known from Iran.

## Materials and methods

The material used for this study was collected by J. Bojková, T. Soldán, J. Imanpour Namin, and S. Bagheri in April and May 2016–2018, and A. Staniczek, M. Pallmann, R. J. Godunko, and F. Nejat in April and May 2017. All specimens were preserved in 75–96% EtOH and are deposited in the collections of the Biology Centre of the Czech Academy of Sciences, Institute of Entomology, České Budějovice, Czech Republic (**IECA**), State Museum of Natural History, Stuttgart, Germany (**SMNS**) and Natural History Museum and Genetic Resources, Department of Environment, Tehran, Iran (**MMTT_DOE**). Material of other *Caucasiron* species used for the morphological and molecular comparisons was obtained from the collection of IECA. This publication and the nomenclatural acts therein are registered with ZooBank under the LSID urn:lsid:zoobank.org:pub:3297FBE4-111C-4849-9533-225A53F7DB3C.

### Morphological examination

Parts of specimens were mounted on microscopic slides using HydroMatrix (MicroTech Lab, Graz, Austria) mounting medium. In order to remove the muscle tissue for an investigation of the cuticular structures, specimens were left overnight in a 10% solution of NaOH prior to slide mounting. Drawings were made using a stereomicroscope Olympus SZX7 and a microscope Olympus BX41, both equipped with a drawing tube. Photographs were obtained using Leica DFC450 camera fitted with macroscope Leica Z16 APO and folded in Helicon Focus version 5.3 X64. All photographs were subsequently enhanced with Adobe Photoshop CS5. Diagnostic characters for the description of larva were chosen according to Braasch and Soldán (1979) and Braasch (2006). The terminology was used mostly according to Kluge and Novikova (2011) and Kluge (2004, 2015).

### DNA extraction, PCR, sequencing and alignment

Total genomic DNA of the species (4–8 specimens/species) was extracted from legs using the DEP-25 DNA Extraction Kit (TopBio s.r.o., Prague, Czech Republic) according to the manufacturer’s protocol. Mitochondrial cytochrome oxidase subunit I (COI) was sequenced according to Hrivniak et al. (2017). COI sequences of other *Caucasiron* species used for comparisons were obtained from Hrivniak et al. (2017) (GenBank accession nos KY865691–KY865725) and Hrivniak et al. (2019) (GenBank accession nos KY865691–KY865725). Three specimens of E. (C.) iranicus were additionally sequenced. The PCR amplification of COI and reaction volumes was carried out as described in Hrivniak et al. (2017). Sequences were assembled in Geneious 7.0.6 (http://www.geneious.com) and aligned in the same software using the Mafft 7.017 (Katoh et al. 2002) plugin with default settings. Newly obtained sequences are deposited in GenBank with accession numbers (GB) MN856180–MN856198.

### Molecular species delimitation

Species were delimited using the single locus (COI) coalescence based General Mixed Yule Coalescent model (GMYC, Pons et al. 2006; Fusijawa and Barraclough 2013). We used the single-threshold GMYC model as it has been found to outperform the multi-threshold (Fusijawa and Barraclough 2013) and was found to be highly suitable for species delimitation within *Caucasiron* (Hrivniak et al. 2019). The GMYC model identifies independent evolutionary clusters by detecting a threshold value at the transition from interspecific to intraspecific branching patterns (Bryson et al. 2013). A maximum likelihood approach is used to optimize the shift in branching patterns. A likelihood ratio test assesses if the mixed model fits the data significantly better than a null model that assumes a single coalescent process for the entire tree (Pons et al. 2006; Monaghan et al. 2009). Analyses were performed using the SPLITS package for R (http://r-forge.r-project.org/projects/splits). An ultrametric COI gene tree was reconstructed under relaxed molecular clock (uncorrelated lognormal distribution) using BEAST 2 (Bouckaert et al., 2014) on CIPRES Science Gateway 3.3 (Miller et al. 2010). An input file was generated in BEAUti 2. The substitution model was selected by bModelTest (Bouckaert and Drummond 2017) implemented in BEAUti 2 using a model averaging approach. A coalescent constant population tree prior was preferred, because the GMYC null model constitutes a single coalescent cluster (Monaghan et al. 2009; Zaldívar-Riverón et al. 2010; Vuataz et al. 2011). Other settings were default. Two analyses of MCMC chains were run for 50 million generations sampled every 5000 generations. Convergence and effective sample size (ESS > 200) were verified using Tracer 1.6. The first 10% of trees (1000) from each run were discarded as burn-in. The files from both independent runs were combined using LogCombiner 1.8.4. The maximum clade credibility tree was constructed from 18000 trees using TreeAnnotator 1.8.4 with default settings.

Inter- and intra-specific K2P pairwise genetic distances were calculated in MEGA 7 (Kumar et al. 2016). The distance matrix was analysed using Automatic Barcode Gap Discovery (ABGD) (Puillandre et al. 2012) (online version: http://wwwabi.snv.jussieu.fr/public/abgd/) with default settings. The method identifies so-called barcode gap that corresponds to threshold between intra- and inter-specific genetic distances and splits sequences to groups corresponding to putative species accordingly.

## Results and discussion

### Taxonomy

All of the species described below are attributed to the subgenusCaucasiron within the genus *Epeorus* based on the presence of projections on the costal rib of gill plates II–VII, and the presence of medio-dorsally directed hair-like setae located on the anterior margin of the head (see Kluge 2015 for a revision of the subgenus).

#### Epeorus (Caucasiron) alborzicus

Taxon classificationAnimaliaEphemeropteraHeptageniidae

Hrivniak & Sroka sp. nov.

622A6F8D-BAD9-5BBA-AC28-119F4D70AD06

http://zoobank.org/F1721BB2-DC7C-4BBC-9AD2-8252A5D01EBF

Figures 1 , 2

##### Type material.

***Holotype***: female mature larva: IRAN, Mazandaran Province, Panjab village, unnamed brook (LT of Haraz River); 36°05'52.8"N, 052°15'16.0"E (locality no. 152); 955 m a.s.l.; J. Bojková, T. Soldán, J. Imanpour Namin, S. Bagheri leg., 9.5.2018, SMNS_EPH_010056.

***Paratypes***: 38 female larvae (3 mounted on slide), 10 male larvae (2 mounted on slide): same data as holotype, SMNS_EPH_010056. DNA extracted from 1 female (code: IR11, stored in EtOH) and 2 males (codes: IR12 and IR14, both stored in EtOH).

33 female larvae, 24 male larvae: IRAN, Tehran Province, Zayegan village, Lalan River; 35°58'39.2"N, 051°34'56.5"E (locality no. 55); 2290 m a.s.l.; A. Staniczek, M. Pallmann, R. J. Godunko, F. Nejat leg., 8.5.2017, SMNS_EPH_007617.

1 female larva: IRAN, Golestan Province, above Chah-e Ja village, unnamed brook (RT of river flowing to Fazelabad); 36°40'22.8"N, 054°46'37.9"E (locality no. 104); 1450 m a.s.l.; J. Bojková, T. Soldán, J. Imanpour Namin leg., 27.4.2018. DNA extracted specimen (code: IR13, stored in EtOH).

17 female larvae (3 mounted on slide), 6 male larvae: IRAN, Alborz Province, 2.5 km W of Asara village, Karaj River; 36°01'52.1"N, 051°13'10.0"E (locality no. 58); 1890 m a.s.l.; A. Staniczek, M. Pallmann, F. Nejat leg., 10.5.2017, SMNS_EPH_007627.

The holotype and 50 paratypes are deposited in SMNS, 50 paratypes (including DNA extracted specimens) are deposited in IECA and 29 paratypes in MMTT_DOE.

##### Other material examined.

8 larvae: same data as holotype, SMNS_EPH_010056; young instars or damaged specimens.

13 larvae: IRAN, Mazandaran Province, NE of Kahrud village, unnamed brook (LT of Haraz River); 36°03'42.7"N, 052°15'24.8"E (locality no. 153); 1020 m a.s.l.; J. Bojková, T. Soldán, J. Imanpour Namin, S. Bagheri leg., 9.5.2018.

2 larvae: IRAN, Mazandaran Province, 3.5 km E of Polour village, Lasem Rud (RT of Haraz River); 35°50'09.4"N, 052°04'38.4"E (locality no. 73); 2100 m a.s.l.; A. Staniczek, M. Pallmann, F. Nejat leg., 14.5.2017, SMNS_EPH_007680; 17 larvae: S. Bagheri leg., 16.4.2018.

1 larva: IRAN, Mazandaran Province, 1.5 km S of Part Kola village, Shirin Rud (LT of Sefidrud); 36°9'04.3"N, 053°20'54.7"E (locality no. 63); 750 m a.s.l.; A. Staniczek, M. Pallmann, F. Nejat leg., 11.5.2017, SMNS_EPH_007641; 10 larvae: S. Bagheri leg., 5.4.2018.

7 larvae: IRAN, Mazandaran Province, 3.5 km W of Razan village, Baladeh River; 36°11'39.6"N, 052°8'34.6"E (locality no. 73); 1360 m a.s.l.; A. Staniczek, M. Pallmann, F. Nejat leg., 14.5.2017, SMNS_EPH_007677.

1 larva: IRAN, Tehran Province, Lalan village, Lalan River; 35°59'50.3"N, 051°34'51.0"E (locality no. 53); 2438 m a.s.l.; A. Staniczek, M. Pallmann, R. J. Godunko, F. Nejat leg., 8.5.2017, SMNS_EPH_007613.

17 larvae: IRAN, Tehran Province, Igol village, Fasham River; 35°55'11.2"N, 051°28'51.3"E (locality no. 56); 2020 m a.s.l.; A. Staniczek, M. Pallmann, R. J. Godunko, F. Nejat leg., 8.5.2017, SMNS_EPH_007618.

10 larvae: IRAN, Alborz Province, 4 km NW of Shahrestanak village, Shahrestanak River; 35°59'01.2"N, 051°19'09.6"E (locality no. 57); 2100 m a.s.l.; A. Staniczek, M. Pallmann, F. Nejat leg., 10.5.2017, SMNS_EPH_007622.

##### Etymology.

The species name refers to the type locality and distribution of the species in the Alborz mountain range.

##### Localities and habitat preferences of larvae.

Larvae inhabit small streams (2–8 m width, 20–50 cm depth) at high altitudes (six of eleven localities at approx. 2000 m a.s.l.) in the central Alborz (Fig. 9). One larva was found in the eastern Alborz (Fig. 9). Larvae were found only in cold and clear streams where they dwelled on large stones in riffles with very fast flow. All localities were situated in deep valleys with rivers draining high mountains. They were mostly treeless, only sometimes with sparse solitary shrubs and trees at the banks (Fig. 10A, B). Streams had a very coarse bed substrate with prevailing boulders and stones and a low share of fine sediments, and turbulent to strongly turbulent flow. They were characteristic of high fluctuation of discharge, with sudden peaks of discharge after spates on the mountains (Fig. 10A).

##### Description of larva.

General colouration of larvae yellowish brown with dark brown maculation. Body length of mature larvae: 13.3–15.8 mm (female), 10.3–11.3 mm (male). Length of cerci approximately 1.3× body length.

**Figure 1.**
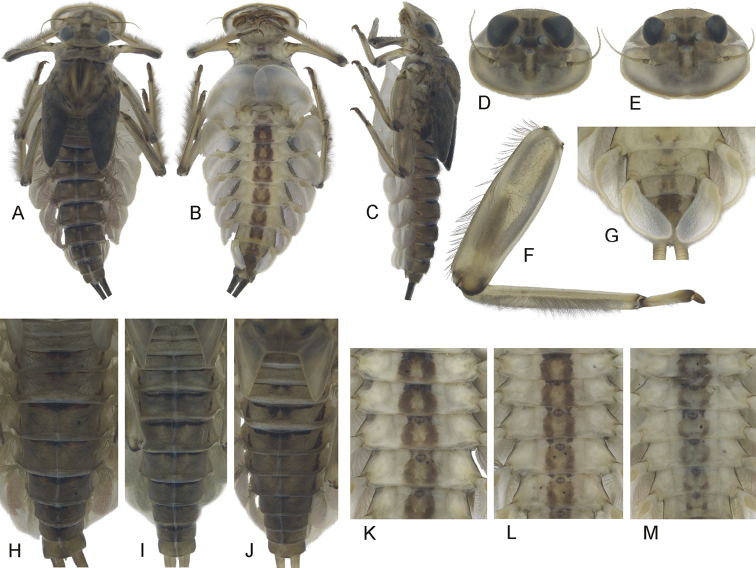
Epeorus (Caucasiron) alborzicus sp. nov., larva: **A** habitus in dorsal view **B** habitus in ventral view **C** habitus in lateral view **D** head of male in dorsal view **E** head of female in dorsal view **F** middle leg in dorsal view **G** distal part of abdomen in ventral view **H–J** colouration of abdominal terga **K–M** colouration of abdominal sterna.

***Head.*** Shape trapezoidal; anterior and lateral margin rounded, posterior margin rounded in female, slightly rounded or nearly straight in male (Fig. 1D, E). Anterior margin with shallow concavity medially. Head dimensions of mature larvae: length 2.8–3.1 mm, width 4.0–4.6 mm (female); length 2.2–2.7 mm, width 3.2–3.7 mm (male). Head width/length ratio: 1.4–1.5 (both male and female). Dorso-medial part with pair of stripes. Pair of maculae located between ocelli (sometimes fused into single macula). Rounded maculae ventrolateral of lateral ocelli and blurred maculae near inner edges of compound eyes. Pale stripes extending horizontally from lateral ocelli to lateral edges of head. Pair of elongated, curved maculae located along coronal suture. Compound eyes grey to black in female, brownish or greyish and basally black in male mature larva. Ocelli blackish, basally paler. Antennae yellowish brown, scapus and pedicellus darkened. Anterior margin of head densely covered with hair-like setae extending to lateral margins and directed medio-dorsally. Dorsal surface of head covered with fine hair-like setae and sparsely distributed stick-like setae. Sparse longer and fine hair-like setae located posteriorly to eyes.

**Figure 2.**
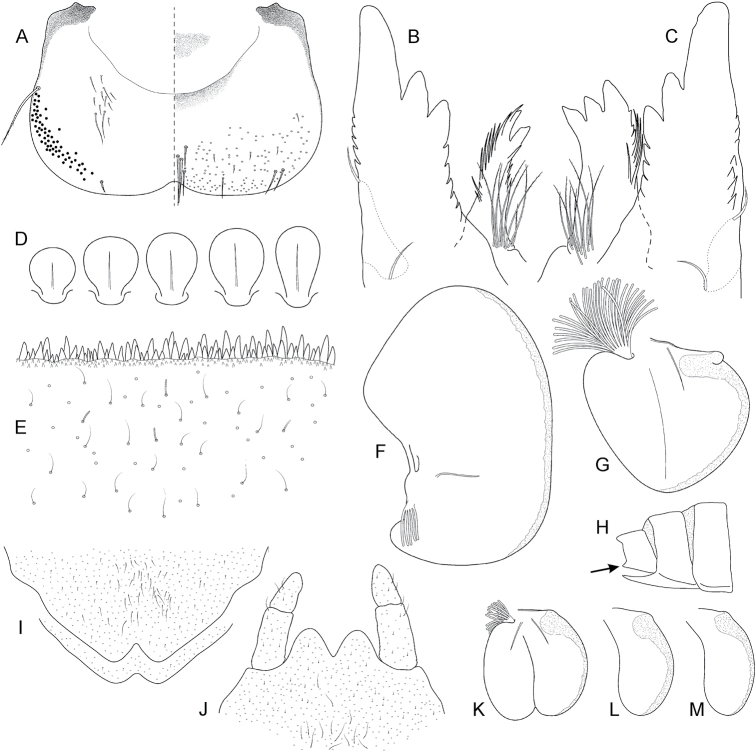
Epeorus (Caucasiron) alborzicus sp. nov., larva: **A** labrum (right half in dorsal view, left half in ventral view) **B** incisors of right mandible in ventral view **C** incisors of left mandible in ventral view (both flattened on slide) **D** setae on dorsal surface of femora **E** surface and posterior margin of abdominal tergum VII **F** gill I **G** gill III **H** abdominal segments VIII–X **I** sternum IX, female **J** sternum IX, male **K** gill VII (flattened on slide) **L–M** gill VII (in natural position from ventral view), variability in shape.

***Mouthparts*.** Labrum (Fig. 2A) widened anteriorly, with anterior margin slightly rounded or nearly straight (in dorsal view). Lateral angles rounded (shape of labrum may vary among individual specimens). Dorsal surface (Fig. 2A, right half) sparsely covered with setae of different size; 4–6 longer bristle-like setae located antero-medially and two bristles antero-laterally. Epipharynx with longer, slightly plumose bristles situated along lateral to anterior margin (Fig. 2A, left half, range of setation figured as large black dots), and cluster of fine, hair-like setae medially (not figured). Posterior margin of labrum irregularly concave; group of 6–17 setae of various size located on ventral surface close to posterior margin. Outer incisors of both mandibles (Fig. 2B, C) with three apical teeth; outer tooth blunt in both mandibles. Inner incisor of left mandible with three apical teeth, right inner incisor bifurcated.

***Thorax*.** Pronotum anteriorly narrowed, lateral edges nearly straight. Metanotum with slight postero-medial projection. Dorsal surface covered with fine, hair-like setae (as on abdominal terga and head); sparse longer, hair-like setae along pro-, meso- and metanotal suture.

***Legs*.** Colour pattern of femora as in Fig. 1F. Femora without medial hypodermal spot. Patella-tibial suture darkened; tarsi proximally and distally darkened. Coxal projections of fore- and hind legs pointed or bluntly pointed; in middle legs blunt. Trochanteres with spatulate setae as on dorsal surface of femora (Fig. 2D). Tibiae of forelegs 1.20–1.37× femur length, tibiae of middle legs 1.0–1.2× femur length, and tibiae of hind legs 0.92–1.08× femur length. Tarsi of all legs 0.26–0.34× tibia length. Dorsal surface of femora covered by short and sporadically elongated spatulate setae (Fig. 2D), hair-like setae, and sparsely distributed stick-like setae. Anterior margin of femora with short, pointed or bluntly pointed spine-like setae; posterior margin with row of long blade-like setae and sparse row of bluntly pointed, spine-like setae. Dorsal margin of tibiae and tarsi with row of long setae; ventral margin of both with irregular row of spine-like setae accumulated distally. Tarsal claws with 2–3 denticles.

***Abdominal terga*.** Colour pattern of abdominal terga (Fig. 1A, H–J) consists of transversal stripe along anterior margin of terga I–IX (X), medially extending to single blurred macula or pair of rounded maculae on terga II–IV and short triangular or nearly rectangular macula on terga V–IX. Terga VIII and IX (X) medially darkened. Pattern of abdominal terga sometimes poorly expressed, only with medially thickened transversal stripe along anterior margin.

Lateral margins with oblique maculae on terga I–IX, sometimes dorso-posteriorly extended. Pair of sigilla sometimes coloured, in form of short stripes or spots located antero-laterally to medial macula. Denticles on posterior margin on terga of various size, irregular and pointed (Fig. 2E). Surface of terga covered with hair-like setae and sparsely with stick-like setae. Tergum X with distinct postero-lateral projections (Fig. 2H, arrow). Supra-tergalial projection (sensu Kluge 2004) short and blunt. Longitudinal row of hair-like setae along abdominal terga present medially.

***Abdominal sterna*.** Yellowish, with distinct colour pattern in form of medial circular macula (Fig. 1B, G, K–M, best expressed on sterna II–VI). Medio-anterior sigilla partly pigmented, lateral sigilla not pigmented; medio-posterior sigilla in form of pale spots in intensively pigmented specimens. Nerve ganglia occasionally darkened. Intensity of colouration varies among individuals (Fig. 1K–M). Sternum IX with V-shaped medial emargination; surface covered by irregularly distributed short hair-like setae, and medially accumulated longer hair-like setae (Fig. 2I, J).

***Gills*.** Dorsal surface of gill plate I yellowish; of gill plates II–VII greyish on anterior half, brownish (sometimes reddish) on posterior half. Ventral margin of all gill plates yellowish. Projection of gill plate III well developed (Fig. 2G). Gill plate VII relatively wide (in natural position of ventral view, Figs 1G, 2L, M). Filaments of gills II–VI reaching 0.40–0.58× length of respective plate, filaments of gill VII reaching 0.18–0.24× (in late-instar larvae).

***Cerci*.** Yellowish brown, basally darkened.

##### Subimago, imago and eggs.

Unknown.

##### Morphological diagnostics of larvae.

The main larval diagnostic characters of E. (C.) alborzicus sp. nov. are as follows: (i) colour pattern of abdominal terga (Fig. 1A, H–J) and sterna (Fig. 1B, K–M), (ii) presence of distinct postero-lateral projections on tergum X (Fig. 2H), (iii) absence of medial hypodermal femur spot (Fig. 1F), (iv) gill plate VII relatively wide (in natural position from ventral view; Figs 1G, 2L, M), and (v) fine hair-like setae on surface of abdominal terga (Fig. 2E).

##### Affinities.

The combination of diagnostic characters mentioned above clearly distinguish larvae of E. (C.) alborzicus sp. nov. from all other *Caucasiron* species known so far. However, some of the diagnostic characters occur also in other *Caucasiron* species distributed in the Caucasus. The colour pattern of abdominal sterna in E. (C.) alborzicus sp. nov. is similar in E. (C.) bicolliculatus (Hrivniak et al. 2017: 356, fig. 8) and E. (C.) alpestris (Braasch 1979: 284, fig. 1d). Both species also lack a medial hypodermal femur spot. Epeorus (C.) bicolliculatus can be distinguished from E. (C.) alborzicus sp. nov. by (i) the presence of flattened setae on the surface of abdominal terga (Hrivniak et al. 2017: 359, fig. 23), (ii) the presence of paired postero-medial protuberances on terga II–IX (Hrivniak et al. 2017: 356, figs 10, 11; 360, figs 31, 32), and (iii) the absence of a postero-lateral projection on the tergum X.

Epeorus (C.) alpestris differs by the characteristic colour pattern of abdominal terga (Braasch 1979: 294, fig. 1c) and the absence of postero-lateral projections on the tergum X.

The presence of postero-lateral projections on the abdominal tergum X is characteristic for two species distributed in the Caucasus, E. (C.) magnus, E. (C.) nigripilosus, and sporadically also in E. (C.) znojkoi. Epeorus (C.) magnus differs from E. (C.) alborzicus sp. nov. in the absence of colouration of abdominal sterna and the characteristic setation on the dorsal margin of labrum (numerous thickened bristle-like setae, Hrivniak et al. in prep.). Epeorus (C.) nigripilosus can be separated from E. (C.) alborzicus sp. nov. by the presence of the distinct medial hypodermal femur spot and unique colour pattern of abdominal sterna (Sinitshenkova 1976: 89, fig. 28). Epeorus (C.) znojkoi can be clearly distinguished from E. (C.) alborzicus sp. nov. by the colour pattern of abdominal terga and conspicuous reddish colouration of abdominal sterna (Braasch 1980: 172, fig. 4b–c).

Two species, E. (C.) soldani and E. (C.) sinitshenkovae, are lacking a medial hypodermal femur spot just like E. (C.) alborzicus sp. nov. Both can be separated from the latter by the absence of postero-lateral projections on tergum X, narrower gill plates VII (in natural position from ventral view), and the absence of a distinct colour pattern of abdominal sterna. Additionally, E. (C.) soldani differs from E. (C.) alborzicus sp. nov. by the presence of flattened setae on the surface of abdominal terga (Hrivniak et al. 2017: 359, fig. 25).

Other *Caucasiron* species distributed in the Caucasus and adjacent areas do not share important diagnostic characters with E. (C.) alborzicus sp. nov. All of these species can be easily distinguished by the following combination of characters: (i) absence of the colour pattern of abdominal sterna and presence of the medial hypodermal femur spot in E. (C.) turcicus, E. (C.) longimaculatus, E. (C.) shargi sp. nov. and (ii) colour pattern of abdominal terga and sterna in E. (C.) caucasicus (Braasch 1979: fig. 3a), E. (C.) iranicus (Braasch and Soldán 1979: fig. 12), and E. (C.) zagrosicus sp. nov. (Fig. 5A–C, G, H–K). The larva of E. (C.) insularis is currently not described.

**Figure 3.**
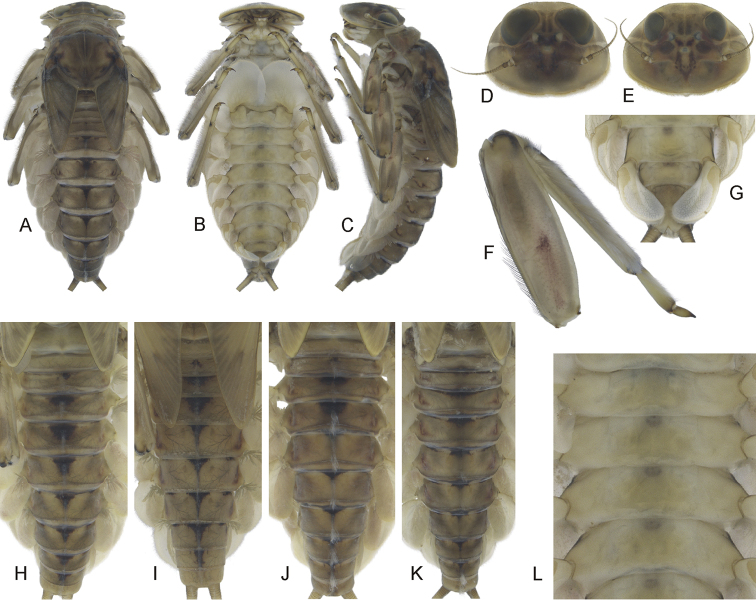
Epeorus (Caucasiron) shargi sp. nov., larva: **A** habitus in dorsal view **B** habitus in ventral view **C** habitus in lateral view **D** head of male in dorsal view **E** head of female in dorsal view **F** middle leg in dorsal view **G** distal part of abdomen in ventral view **H–K** colouration of abdominal terga **L** colouration of abdominal sterna.

#### Epeorus (Caucasiron) shargi

Taxon classificationAnimaliaEphemeropteraHeptageniidae

Hrivniak & Sroka sp. nov.

2733384A-E11D-572D-9F51-75256E2F2AF3

http://zoobank.org/6F5FE6F7-8710-416D-80DB-C202C71DE7FC

Figures 3 , 4

##### Type material.

***Holotype***: female mature larva: IRAN, Golestan Province, Shirinabad village, unnamed river; 36°48'01.4"N, 055°01'05.8"E (locality no. 108); 740 m a.s.l.; J. Bojková, T. Soldán, J. Imanpour Namin leg., 27.4.2018, SMNS_EPH_010057.

***Paratypes***:

19 female, 11 male larvae: same data as holotype.

36 female (5 mounted on slide), 25 male (1 mounted on slide) larvae: IRAN, Golestan Province, above Chah-e Ja village, unnamed brook (RT of river flowing to Fazelabad); 36°40'22.8"N, 054°46'37.9"E (locality no. 104); 1450 m a.s.l.; J. Bojková, T. Soldán, J. Imanpour Namin leg., 27.4.2018. DNA extracted from 2 females (codes: IR23 and IR24, mounted on slides).

19 female (3 mounted on slide), 7 male (1 mounted on slide) larvae: IRAN, Golestan Province, below Chah-e Ja village (main valley), unnamed river flowing to Fazelabad, 36°41'46.3"N, 054°47'35.0"E (locality no. 105); 1240 m a.s.l.; J. Bojková, T. Soldán, J. Imanpour Namin leg., 27.4.2018. DNA extracted from 1 female (code: IR21, mounted on slide) and 1 male (code: IR22, stored in EtOH).

The holotype (SMNS_EPH_010057) and 50 paratypes (SMNS_EPH_010057) are deposited in SMNS, 50 paratypes (including DNA extracted specimens) are deposited in IECA, and 17 paratypes in MMTT_DOE.

##### Other material examined (not paratypes):

3 larvae: same data as holotype; young instars or damaged specimens.

##### Etymology.

The species name derives from *shargi* (یقرش), which means eastern in Farsi. It refers to the distributional range of the species in the eastern part of the Alborz mountain range.

##### Localities and habitat preferences of larvae.

Larvae were found in three clear streams at middle altitude (740–1450 m a.s.l.) in the eastern Alborz (Fig. 9). Habitat conditions of these streams differed from each other. Larvae were abundant in a cold, alkaline brook (water conductivity 1320 μS/cm) with patches of precipitated calcium crusts on the bed and in the non-alkaline water (with water conductivity reaching the values of clear montane streams in the region, 433 μS/cm) of the type locality. Both localities were characterised by stony bed sediment with leaf litter debris and fine gravel along the banks, and by fast, turbulent flow (Fig. 10C, D). Lower abundance of larvae was found in a river with uniform coarse substrate flowing in a wide gravel river channel. All streams were surrounded by deciduous forests (Fig. 10C, D). The species was not found in urban and agricultural areas in this region where many localities were investigated.

##### Description of larva.

General colouration of larvae yellowish brown with dark brown maculation. Body length of mature larvae 13.7–15.6 mm (female), 11.7–13.0 mm (male). Length of cerci approximately 1.1× body length.

***Head.*** Shape trapezoidal; anterior and lateral margin rounded, posterior margin rounded in female, slightly rounded in male (Fig. 3D, E). Anterior margin with shallow concavity medially. Head dimensions of mature larvae: length 3.0–3.2 mm, width 4.1–4.4 mm (female); length 2.70–2.95 mm, width 3.5–4.0 mm (male). Head width/length ratio: 1.33–1.40 (both male and female). Dorso-medial part with brown, rectangular or oval smudge, sometimes reduced to pair of stripes. Pair of maculae located between ocelli (sometimes fused into single macula). Rounded maculae lateroventral of lateral ocelli and blurred maculae near inner edges of compound eyes. Pair of pale stripes extending from lateral ocelli to lateral edges of head. Pair of maculae located along coronal suture. Compound eyes dark grey to black in female, brownish and basally blackish in male mature larva. Ocelli dark grey to black, basally paler. Antennae yellowish-brown, scapus and pedicellus darkened. Anterior margin of head densely covered with hair-like setae extending to lateral margins and directed medio-dorsally. Dorsal surface of head covered with fine hair-like setae and sparsely distributed stick-like setae. Sparse longer fine hair-like setae located posteriorly to eyes.

**Figure 4.**
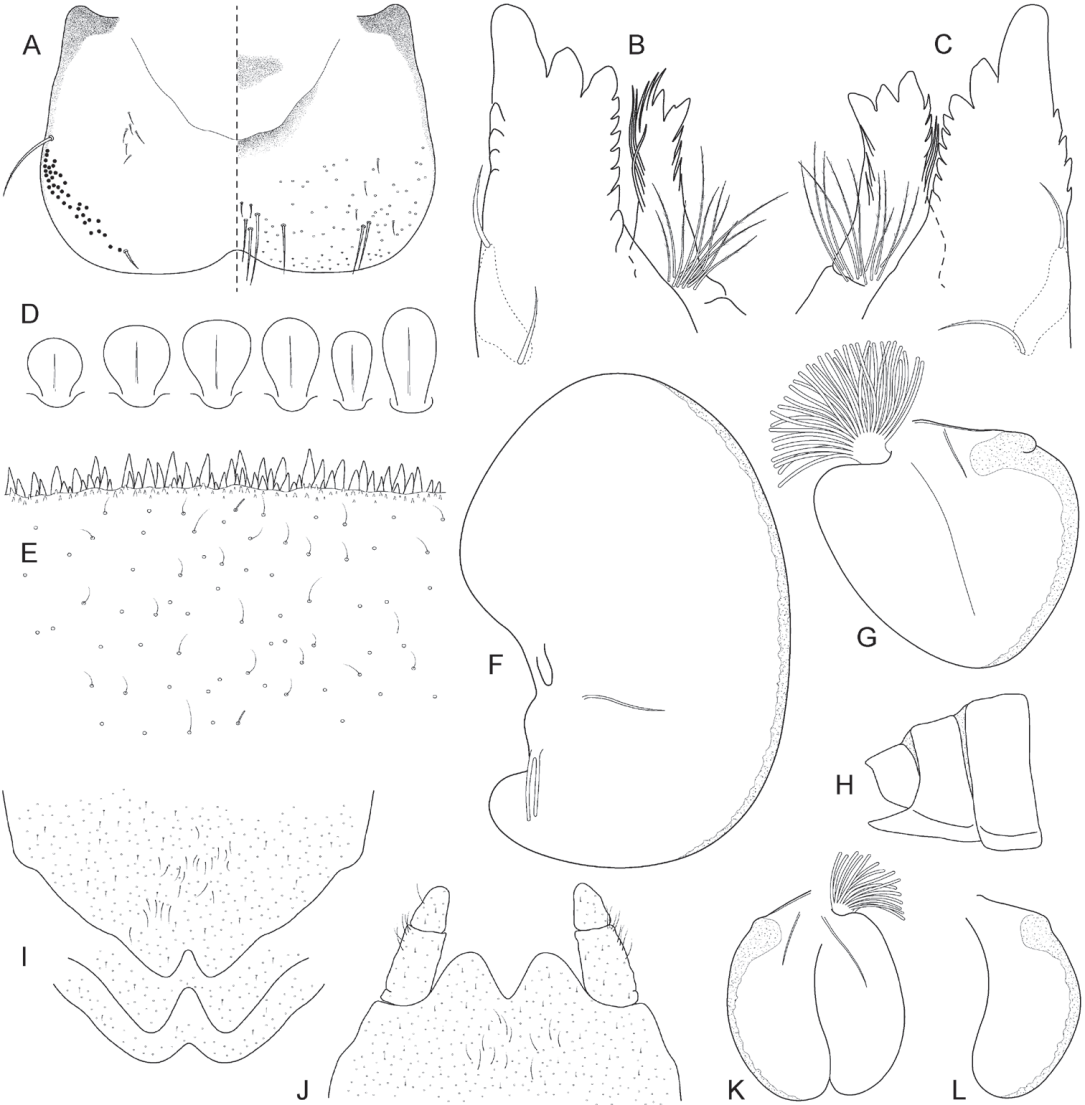
Epeorus (Caucasiron) shargi sp. nov., larva: **A** labrum (right half in dorsal view, left half in ventral view) **B** incisors of right mandible in ventral view **C** incisors of left mandible in ventral view (both flattened on slide) **D** setae on dorsal surface of femora **E** surface and posterior margin of abdominal tergum VII **F** gill I **G** gill III **H** abdominal segments VIII–X **I** sternum IX, female **J** sternum IX, male **K** gill VII (flattened on slide) **L** gill VII (in natural position from ventral view).

***Mouthparts*.** Labrum (Fig. 4A) widened anteriorly, with anterior margin slightly rounded or nearly straight (in dorsal view). Lateral angles rounded (shape of labrum may vary among individual specimens). Dorsal surface (Fig. 4A, right half) sparsely covered with setae of different size; 4–6 longer bristle-like setae located antero-medially and two antero-laterally. Epipharynx with longer, shortly plumose bristles situated along lateral to anterior margin (Fig. 4A, left half), range of setation figured as large black dots), and brush of fine hair-like setae medially (not figured). Posterior margin of labrum irregularly concave; with group of 5–10 setae of various size located on ventral surface close to posterior margin. Outer incisors of both mandibles (Fig. 4B, C) with three apical teeth; outer tooth blunt in both mandibles. Inner incisor of left mandible with three apical teeth, right inner incisor bifurcated (inner side of right tooth usually with small denticle).

***Thorax.*** Pronotum anteriorly narrowed, lateral edges nearly straight or slightly rounded. Metanotum with slight, blunt, postero-medial projection. Dorsal surface covered with fine hair-like setae (as on abdominal terga and head); sparse longer hair-like setae along pro-, meso- and metanotal suture.

***Legs.*** Colour pattern of femora as in Fig. 3F. Femora with rounded or slightly elongated medial hypodermal femur spot. Patella-tibial suture darkened; tarsi proximally and distally darkened. Coxal projections of fore- and hind legs pointed or bluntly pointed; of middle legs blunt. Trochanteres with spatulate setae as on dorsal surface of femora (Fig. 4D). Tibiae of forelegs 1.23–1.28× femur length, tibiae of middle legs 1.03–1.50× femur length, and tibiae of hind legs 0.87–1.06× femur length. Tarsi of all legs 0.28–0.32× tibia length. Dorsal surface of femora covered by short, sporadically elongated spatulate setae (Fig. 4D), hair-like setae, and sparsely distributed stick-like setae. Anterior margin of femora with short, pointed and/or bluntly pointed spine-like setae; posterior margin with row of long blade-like setae and sparse row of bluntly pointed spine-like setae. Dorsal margin of tibiae and tarsi with row of long setae; ventral margin of both with irregular row of spine-like setae accumulated distally. Tarsal claws with 2–3 denticles.

***Abdominal terga.*** Colour pattern of abdominal terga (Fig. 3A, H–K) consists of transversal stripe along anterior margin of terga I–IX (X) medially extending to i) triangular or blurred macula on terga (II) III–IV; ii) triangular or T-shaped macula on terga V–IX, reaching to half or stretching to posterior margin of corresponding tergum (medial macula of terga VIII and IX often widened). Transversal stripe along anterior margin of terga laterally extends to pair of short maculae. Medial maculae often surrounded by pale background. Tergum X without distinct maculation. Pair of sigilla sometimes coloured and forming pair of short stripes adjacent laterally to medial macula. Lateral margins of abdomen with oblique maculae on terga I–IX. Denticles along posterior margin on terga of various size, irregular and pointed (Fig. 4E). Surface of terga covered with hair-like setae and sparsely with stick-like setae. Supra-tergalial projections short and blunt. Tergum X without distinct postero-lateral projections (Fig. 4H). Longitudinal row of hair-like setae along abdominal terga present medially.

***Abdominal sterna.*** Yellowish, without distinct colour pattern. Nerve ganglia often dark brown pigmented (Fig. 3B, G, L). Sternum IX with V-shaped medial emargination; surface covered by irregularly distributed short hair-like setae and medially accumulated longer hair-like setae (Fig. 4I, J).

***Gills.*** Dorsal surface of gill plate I yellowish, of gill plates II–VII greyish on anterior half and brownish to reddish on posterior half. Ventral margin of all gill plates yellowish. Projection of gill plate III well developed (Fig. 4G). Gill plate VII relatively wide (in natural position of ventral view, Figs 3G, 4L). Gill filaments reaching to 0.41–0.50× length of respective plate, filaments of gill VII to 0.24–0.28× (in late-instar larvae).

***Cerci.*** Brownish, basally darkened.

##### Subimago, imago and eggs.

Unknown

##### Morphological diagnostics of larvae.

The main larval diagnostic characters of E. (C.) shargi sp. nov. are as follows: (i) colour pattern of abdominal terga (Fig. 3A, H–K) and no colouration of abdominal sterna (Fig. 3B, G, L), (ii) lack of distinct postero-lateral projections on tergum X (Fig. 4H), (iii) presence of medial hypodermal femur spot (Fig. 3F), (iv) relatively wide shape of gill plate VII (in natural position from ventral view; Figs 3G, 4L), and (v) fine hair-like setae on surface of abdominal terga (Fig. 4E).

##### Affinities.

Based on the colour pattern of abdominal terga and sterna, E. (C.) shargi sp. nov. resembles several species distributed in the Caucasus and adjacent areas. At first glance, E. (C.) soldani and E. (C.) turcicus are most similar. Larvae of E. (C.) soldani possess triangular maculae on abdominal terga (Braasch 1979: 284, fig. 2b) and an indistinct, sometimes not expressed, colour pattern of abdominal sterna. It can be distinguished from E. (C.) shargi sp. nov. by a comparatively narrower gill plate VII (in natural position from ventral view), the presence of flattened setae on the surface of abdominal terga (Hrivniak et al. 2017: 359, fig. 25), and the absence of a medial hypodermal femur spot.

Epeorus (C.) turcicus shares with E. (C.) shargi sp. nov. the lack of colouration on abdominal sterna (Hrivniak et al. 2019: 61, fig. 2), the presence of a medial hypodermal femur spot (Hrivniak et al. 2019: 62, fig. 9), and fine hair-like setae on the dorsal surface of abdominal terga (Hrivniak et al. 2019: 63, fig. 11). Nevertheless, E. (C.) turcicus differs from E. (C.) shargi sp. nov. by the different colour pattern of abdominal terga, with anteriorly widened stripe stretching between anterior and posterior margins (Hrivniak et al. 2019: 61, fig. 1), in contrast to E. (C.) shargi sp. nov. with more or less triangular maculae on abdominal terga (Fig. 3A, H–K), and a distinctly narrower gill plate VII (in natural position from ventral view) (Hrivniak et al. 2019: 63, figs 15, 16).

Similar to E. (C.) shargi sp. nov., there is no colour pattern of abdominal sterna in several other species, namely E. (C.) longimaculatus, E. (C.) sinitshenkovae, and E. (C.) magnus. Epeorus (C.) longimaculatus can be clearly separated from E. (C.) shargi sp. nov. by (i) a distinctly narrower gill plate VII (in natural position of ventral view), (ii) flattened setae on the surface of abdominal terga (Hrivniak et al. 2017: 359, fig. 25), (iii) poorly developed projection on the costal margin of gill plate III (Braasch 1980: 172, fig. 6b), and (iv) elongated medial hypodermal femur spot (Braasch 1980: 172, fig. 11).

Epeorus (C.) sinitshenkovae can be distinguished from E. (C.) shargi sp. nov. by the absence of a medial hypodermal femur spot, the characteristic colour pattern of femora (Braasch and Zimmerman 1979: 106, fig. 10), and the colour pattern of abdominal terga (Braasch 1979: 105, fig. 2).

Epeorus (C.) magnus can be reliably distinguished by the presence of distinct postero-lateral projections on abdominal tergum X and characteristic setation of labrum (numerous thickened bristle-like setae, Hrivniak et al. in prep.).

**Figure 5.**
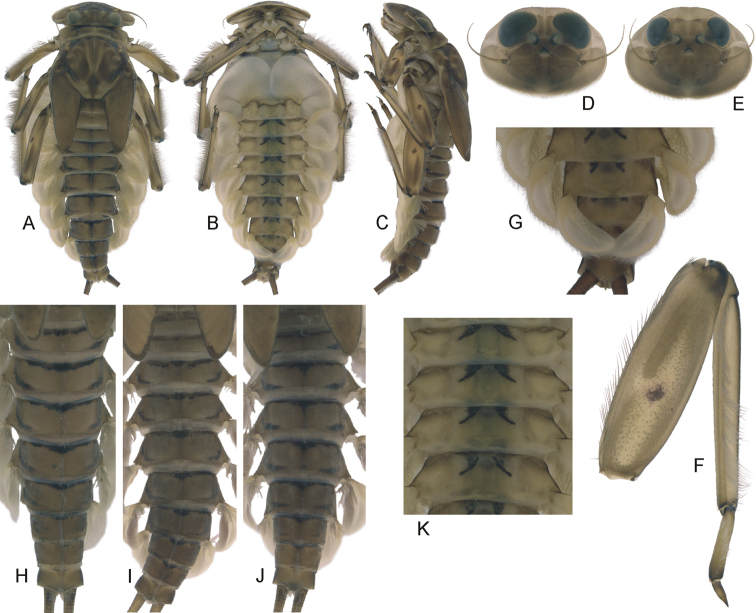
Epeorus (Caucasiron) zagrosicus sp. nov., larva: **A** habitus in dorsal view **B** habitus in ventral view **C** habitus in lateral view **D** head of male in dorsal view **E** head of female in dorsal view **F** middle leg in dorsal view **G** distal part of abdomen in ventral view **H–J** colouration of abdominal terga **K** colouration of abdominal sterna.

All other species distributed in the Caucasus and adjacent areas differ from E. (C.) shargi sp. nov. by the distinct colour pattern of abdominal sterna, namely E. (C.) bicolliculatus (Hrivniak et al. 2017: 356, figs 7–9), E. (C.) alpestris (Braasch, 1979: 284, fig. 1d), E. (C.) alborzicus sp. nov., (Fig. 1B, K–M), E. (C.) caucasicus, E. (C.) iranicus (Braasch 1979: 284, fig. 3b), E. (C.) nigripilosus (Sinitshenkova 1976: 89, fig. 28), E. (C.) znojkoi (Braasch, 1980: 172, 4b), and E. (C.) zagrosicus sp. nov. (Fig. 5B, K).

#### Epeorus (Caucasiron) zagrosicus

Taxon classificationAnimaliaEphemeropteraHeptageniidae

Hrivniak & Sroka sp. nov.

0EADC255-F668-5AC8-A0E5-C7A34C65071C

http://zoobank.org/A49F6070-C918-4FA2-9287-D0B3D9BDBC01

Figures 5 , 6

##### Type material.

***Holotype***: female larva: IRAN, Lorestan Province, 4.5 km SW of Varayeneh village, Sarab-e Gamasiab River, 34°2'46.2"N, 048°22'32.6"E (locality no. 9); 1842 m a.s.l.; A. Staniczek, M. Pallmann, A. Abdoli, F. Nejat leg., 25.4.2017, SMNS_EPH_007520.

***Paratypes***: 79 female larvae, 68 male larvae: same data as holotype, SMNS_EPH_007520. 6 female (2 mounted on slide), 5 male (2 mounted on slide) larvae: IRAN, Chaharmahal and Bakhtiari Province, Dimeh village, Chehme-Dimeh River, 32°30'11.6"N, 050°13'04.5"E (locality no. 45) ; 2220 m a.s.l.; A. Staniczek, M. Pallmann, R. J. Godunko, F. Nejat leg., 5.5.2017, SMNS_EPH_007707. DNA extracted from 3 females (code: IR32, stored in EtOH; codes: IR34 and IR35, mounted on slides) and 2 males (codes: IR33b and IR36, mounted on slides).

15 female (3 mounted on slide), 5 male larvae: IRAN, Kohgiluyeh and Boyer-Ahmad Province, 4 km E of Yasuj, Yasuj fall, 30°40'34.7"N, 051°37'35.6"E (locality no. 37); 2060 m a.s.l.; A. Staniczek, M. Pallmann, R. J. Godunko, F. Nejat leg., 4.5.2017, SMNS_EPH_007568. DNA extracted from 2 females (code: SP38, mounted on slide; code: IR33a, stored in EtOH) and 1 male (code: SP37, stored in EtOH).

2 female, 2 male larvae: IRAN, Chaharmahal and Bakhtiari Province, 5 km W of Chelgerd, Kouhrang River, 32°28'9.3"N, 050°5'26.2"E (locality no. 46); 2402 m a.s.l.; A. Staniczek, M. Pallmann, R. J. Godunko, F. Nejat leg., 5.5.2017, SMNS_EPH_007689.

The holotype and 100 paratypes are deposited in SMNS, 50 paratypes (including DNA extracted specimens) are deposited in IECA and 32 paratypes in MMTT_DOE.

##### Other material examined:

42 larvae: same data as holotype; young instars or damaged specimens.

1 male larva: IRAN, Chaharmahal and Bakhtiari Province, 4 km E. of Bajgiran, Dehno River, 31°54'26.2"N, 050°42'20.6"E (locality no. 50); 1721 m a.s.l.; A. Staniczek, M. Pallmann, R. J. Godunko, F. Nejat leg., 6.5.2017, SMNS_EPH_007606.

##### Etymology.

The species name refers to its known records in the Zagros mountain range.

##### Localities and habitat preferences of larvae.

Larvae were found in five streams of different size at high altitude, above 1700 m a.s.l. Three streams were strongly turbulent rivers with very coarse bed substrate flowing in high-mountain valleys (Fig. 10E). Larvae were found also in a shallow, slow-flowing brook with finer, gravel substrate flowing in the forest (locality near Yasuj fall, Fig. 10F), and in a small stream with moderate, slightly turbulent flow and stony bed substrate with fine gravel, silt, and macrophytes (Chehme-Dimeh River). The species was not found in streams that were polluted or seasonally drying out.

##### Description of larva.

General colouration of larvae yellowish brown with dark brown maculation. Body length of mature larvae 13.5–14.5 mm (female), 10.0–11.0 mm (male). Length of cerci approximately 1.3× body length.

***Head.*** Shape trapezoidal; anterior and lateral margin rounded, posterior margin slightly rounded or nearly straight (Fig. 5D, E). Anterior margin with shallow concavity medially.

Head dimensions of mature larvae: length 2.6–2.7 mm, width 3.6–4.0 mm (female); length 2.3–2.4 mm, width 3.3 mm (male). Head width/length ratio: 1.36–1.49 (both male and female).

Dorso-medial part with indistinct brown rectangular or oval macula, sometimes reduced to pair of stripes. Rounded maculae under lateral ocelli and blurred or triangular maculae near inner edges of compound eyes. Pair of pale stripes extending from lateral ocelli to lateral edges of head. Pair of maculae located along coronal suture. Compound eyes dark grey to black in female, brownish and basally blackish in male mature larva. Ocelli dark grey to black, basally paler. Antennae yellowish-brown, scapus and pedicellus darkened. Anterior margin densely covered with hair-like setae extending to lateral margins and directed medio-dorsally. Dorsal surface covered with fine hair-like setae and sparsely distributed stick-like setae. Sparse longer, fine, hair-like setae located posteriorly to eyes.

**Figure 6.**
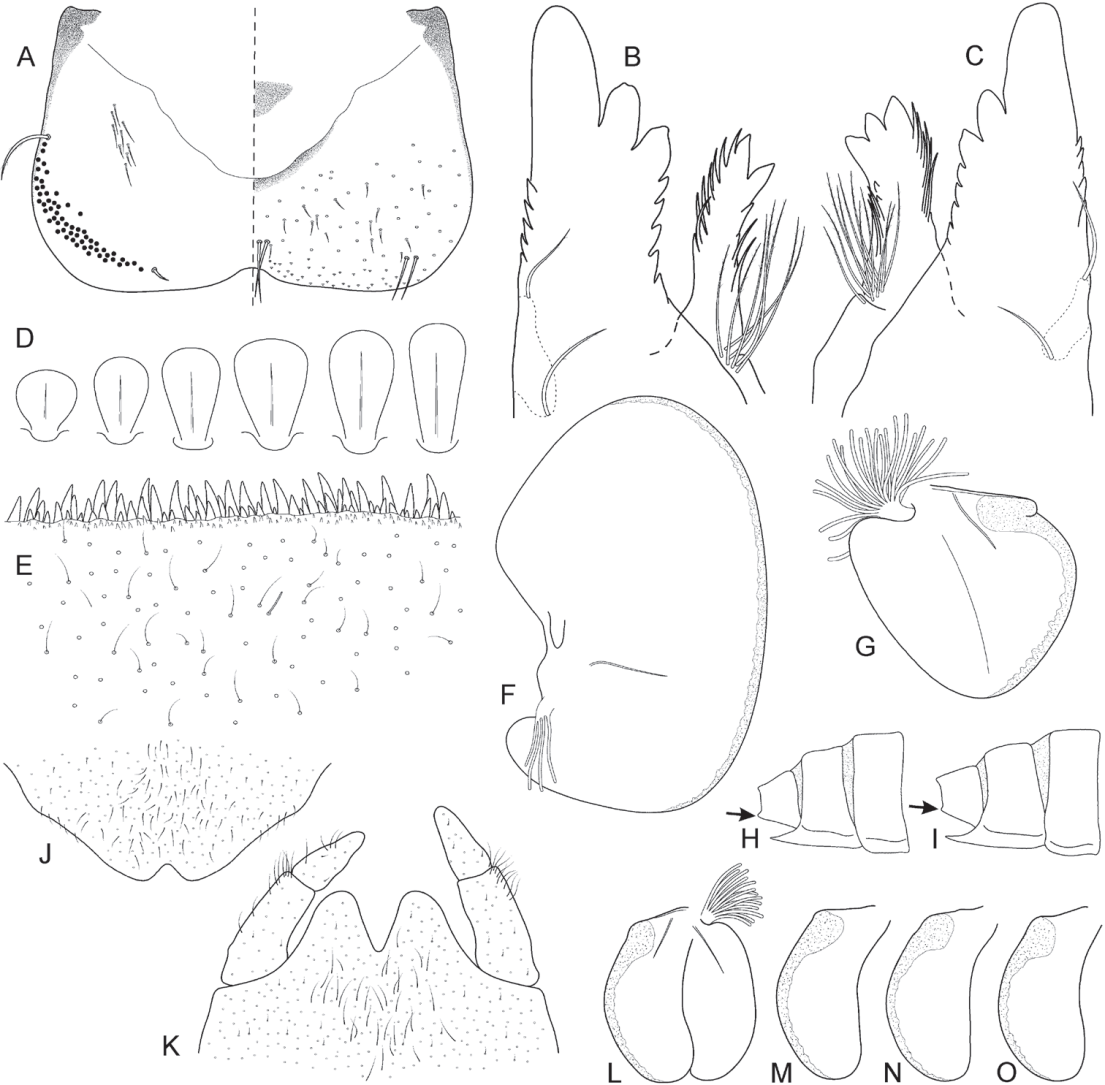
Epeorus (Caucasiron) zagrosicus sp. nov., larva: **A** labrum (right half in dorsal view, left half in ventral view) **B** incisors of right mandible in ventral view **C** incisors of left mandible in ventral view (both flattened on slide) **D** setae on dorsal surface of femora **E** surface and posterior margin of abdominal tergum VII **F** gill I **G** gill III **H–I** abdominal segments VIII–X **J** sternum IX, female **K** sternum IX, male **L** gill VII (flattened on slide) **M–O** gill VII (in natural position from ventral view), variability in shape.

***Mouthparts.*** Labrum (Fig. 6A) widened anteriorly, with anterior margin slightly rounded or nearly straight (in dorsal view). Lateral angles rounded (shape of labrum may vary among individual specimens). Dorsal surface (Fig. 6A, right half) sparsely covered with setae of different size; four longer, bristle-like setae located antero-medially and two antero-laterally. Epipharynx with longer, shortly plumose bristles situated along lateral to anterior margin (Fig. 6A, left half; range of setation figured as large black dots), and brush of fine hair-like setae medially (not figured). Posterior margin of labrum irregularly concave; with group of 6–10 setae of various size located on ventral surface close to posterior margin. Outer incisors of both mandibles (Fig. 6B, C) with three apical teeth; outer tooth blunt in both mandibles. Inner incisor of left mandible with three apical teeth, right inner incisor bifurcated.

***Thorax.*** Pronotum anteriorly narrowed, lateral edges nearly straight. Metanotum with slight postero-medial projection. Dorsal surface covered with fine hair-like setae (as on abdominal terga and head); sparse longer hair-like setae along pro, meso- and metanotal suture.

***Legs.*** Colour pattern of femora as in Fig. 5F. Femora with rounded medial hypodermal femur spot. Patella-tibial suture darkened; tarsi proximally and distally darkened. Coxal projections of fore- and hind legs pointed or bluntly pointed; of middle legs blunt. Trochanteres with spatulate setae as on dorsal surface of femora (Fig. 6D). Tibiae of forelegs 1.20–1.31× femur length, tibiae of middle legs 1.06–1.14× femur length, and tibiae of hind legs 0.90–1.04× femur length. Tarsi of all legs 0.25–0.34× tibia length. Dorsal surface of femora covered by elongated and sporadically short rounded spatulate setae (Fig. 6D); hair-like setae and sparsely distributed stick-like setae. Anterior margin of femora with short, pointed and/or bluntly pointed spine-like setae; posterior margin with row of long blade-like setae and sparse row of bluntly pointed spine-like setae. Dorsal margin of tibiae and tarsi with row of long setae; ventral margin of both with irregular row of spine-like setae accumulated distally. Tarsal claws with two or three denticles.

**Figure 7.**
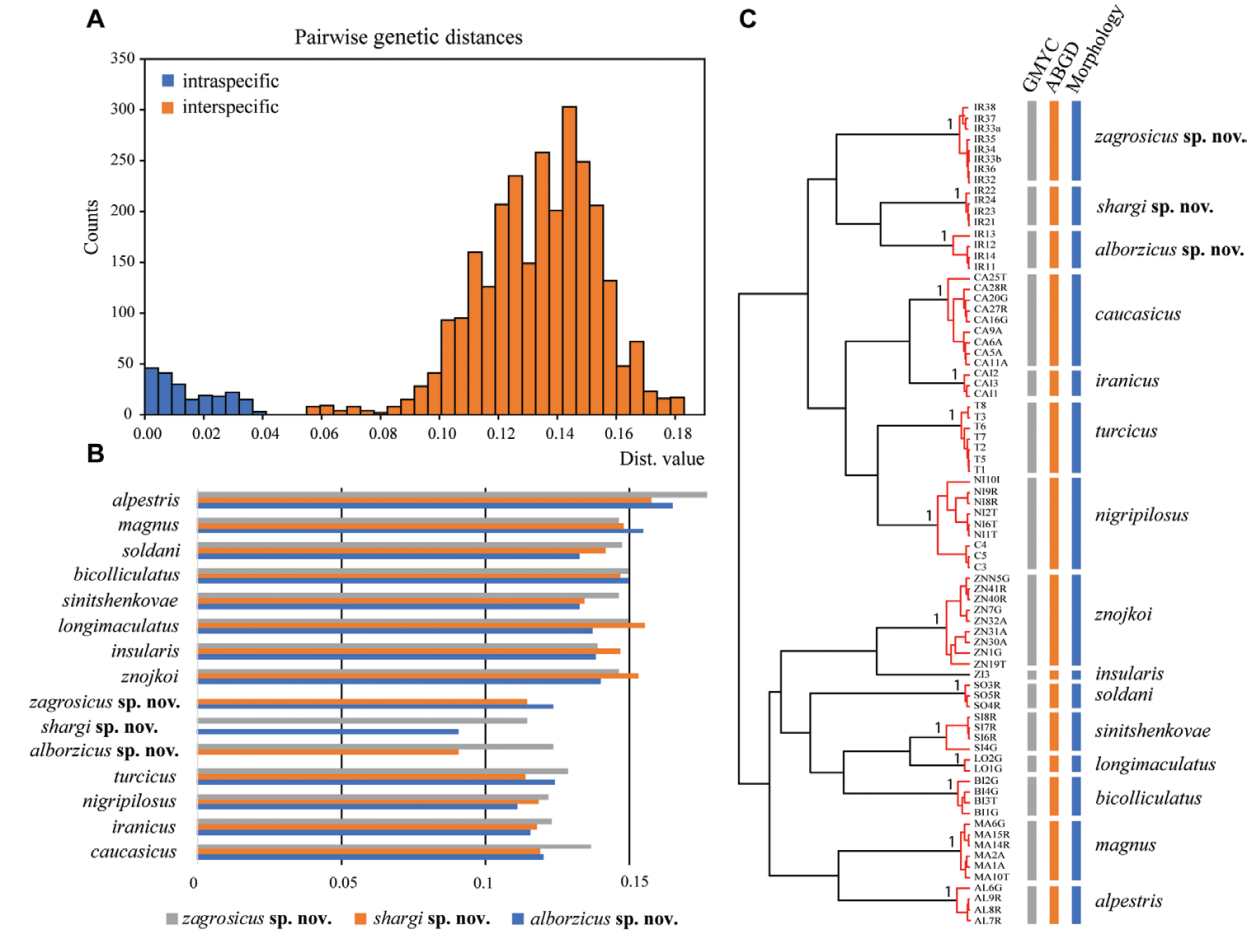
Results of the molecular species delimitation: **A** distribution of K2P pairwise genetic distances **B** mean pairwise genetic distances between new species and all Caucasian *Caucasiron* species known **C**COI gene tree with the results of molecular species delimitation analyses and morphology (node supports for species are indicated).

***Abdominal terga.*** Colour pattern of abdominal terga includes transversal stripe along anterior margin of terga I–IX (X) medially extending to triangular, short rectangular or stripe-like medial macula on terga (III) IV–IX (transversal stripe sometimes not distinctly extended, for variability see Fig. 5A, H–J). Pair of sigilla sometimes coloured, in form of short stripes or spots located antero-laterally to medial macula. Tergum X without distinct maculation. Lateral margins of abdomen with oblique maculae on terga I–IX extending to dorso-posterior margin. Denticles along posterior margin on terga of various size, irregular and pointed, sometimes curved (Fig. 6E). Surface of terga covered with hair-like setae and sparsely with stick-like setae. Supra-tergalial projections short and blunt. Tergum X with more or less developed postero-lateral projections (Fig. 6H, I, arrows). Longitudinal row of hair-like setae medially along abdominal terga present.

***Abdominal sterna.*** Yellowish, with distinct colouration pattern consisting of anteriorly widened pair of stripes (medio-anterior sigilla) on terga II–VIII (Fig. 5B, G, K). Sometimes only oblique stripes are present, without anterior widening (especially on sterna VI–VIII). Nerve ganglia occasionally darkened. Intensity of colouration varies among individuals. Sternum IX with V-shaped medial emargination; surface covered by irregularly distributed short hair-like setae, and medially accumulated longer hair-like setae (Fig. 6J, K).

**Figure 8.**
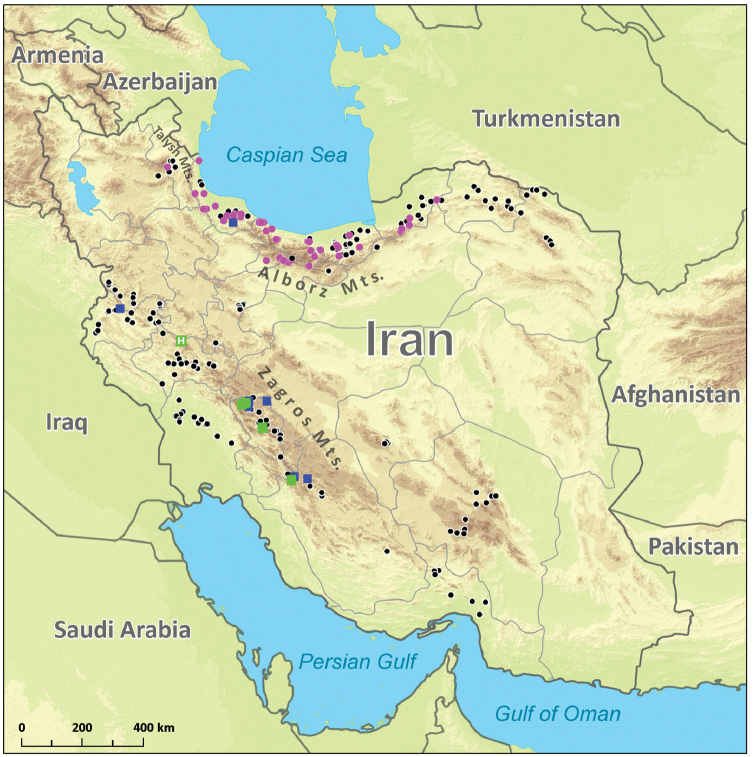
The map showing the occurrence of Epeorus (Caucasiron) spp. at all localities investigated in Iran. Colour of symbols shows the occurrence of species: green – Epeorus (Caucasiron) zagrosicus sp. nov., dark blue – Epeorus (Epeorus) zaitzevi, and violet – all other Epeorus (Caucasiron) species. Black symbols show collection points where no species of *Epeorus* was found. The letter H shows the locality of the respective holotype.

***Gills.*** Dorsal surface of gill plate I yellowish; of gill plates II–VII greyish on anterior half and brownish to reddish on posterior half. Ventral margin of all gill plates yellowish. Projection of gill plate III well developed (Fig. 6G). Shape of gill plate VII (in natural position from ventral view) varies from narrow to relatively wide (Figs 5G, 6M–O). Gill filaments reaching to 0.4–0.5× length of respective plate, filaments of gill VII to 0.24–0.30× (in late-instar larvae).

***Cerci.*** Brownish, basally darkened.

##### Subimago, imago and eggs.

Unknown

##### Morphological diagnostics of larvae.

The main larval diagnostic characters of E. (C.) zagrosicus sp. nov. are as follows: (i) colour pattern of abdominal sterna (Fig. 5B, G, K) and abdominal terga (Fig. 5A, H–J), (ii) presence of postero-lateral projections on tergum X (Fig. 6H, I), (iii) presence of hypodermal medial femur spot (Fig. 5F), and (iv) fine hair-like setae on surface of abdominal terga (Fig. 6E).

**Figure 9.**
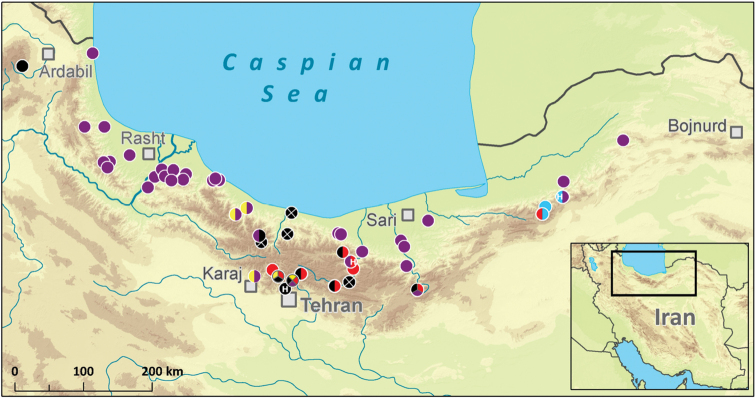
Distribution of Epeorus (Caucasiron) species in northern Iran. Colour of symbols shows the occurrence of species: red – E. (C.) alborzicus sp. nov., light blue – E. (C.) shargi sp. nov., violet – E. (C.) cf. znojkoi, black – E. (C.) iranicus, yellow – E. (C.) nigripilosus. The letter H shows the localities of holotypes. Black symbols with white cross show unrevised records of E. (C.) iranicus.

##### Affinities.

Based on the colour pattern of abdominal sterna, E. (C.) zagrosicus sp. nov. is most similar to E. (C.) caucasicus and E. (C.) iranicus. Both latter species possess pigmented medio-anterior sigilla forming a pair of oblique stripes on abdominal sterna II–VIII (e.g., Braasch 1979: 284, fig. 3b), and a medial hypodermal femur spot. However, E. (C.) zagrosicus sp. nov. differs by the distinct widening at the anterior margin of medio-anterior sigilla of abdominal sterna. If the sternal colour pattern is not fully developed (sporadically only stripes are present on all or several sterna), E. (C.) zagrosicus sp. nov. is distinguishable by the colour pattern of abdominal terga (Fig. 5A, H–J), which is different in E. (C.) caucasicus (Braasch 1979: 284, fig. 3a) and E. (C.) iranicus (Braasch and Soldán 1979: 264, fig. 12). In E. (C.) zagrosicus sp. nov., the postero-lateral projections on the tergum X are well-developed, whereas they are not significantly pronounced in either of the two species mentioned above (only small projections may be sporadically present).

Distinct postero-lateral projections on the tergum X are characteristic for E. (C.) magnus, E. (C.) nigripilosus, and E. (C.) alborzicus sp. nov. Small projections are also sporadically present in E. (C.) znojkoi. E. (C.) magnus can be easily distinguished from E. (C.) zagrosicus sp. nov. by the absence of colour pattern of abdominal sterna, the absence of a medial hypodermal femur spot, and setation on dorsal margin of labrum (numerous thickened bristle-like setae, Hrivniak et al., in prep.). E. (C.) nigripilosus and E. (C.) alborzicus sp. nov. differ by a typical colouration pattern of abdominal sterna (Sinitshenkova 1976: 89, fig. 28 for E. (C.) nigripilosus and Fig. 1B, G, K–M for E. (C.) alborzicus sp. nov.). E. (C.) znojkoi can be distinguished from E. (C.) zagrosicus sp. nov. by the colour pattern of abdominal terga and characteristic reddish colouration of abdominal sterna (Braasch 1980: 172, fig. 4b, c).

**Figure 10.**
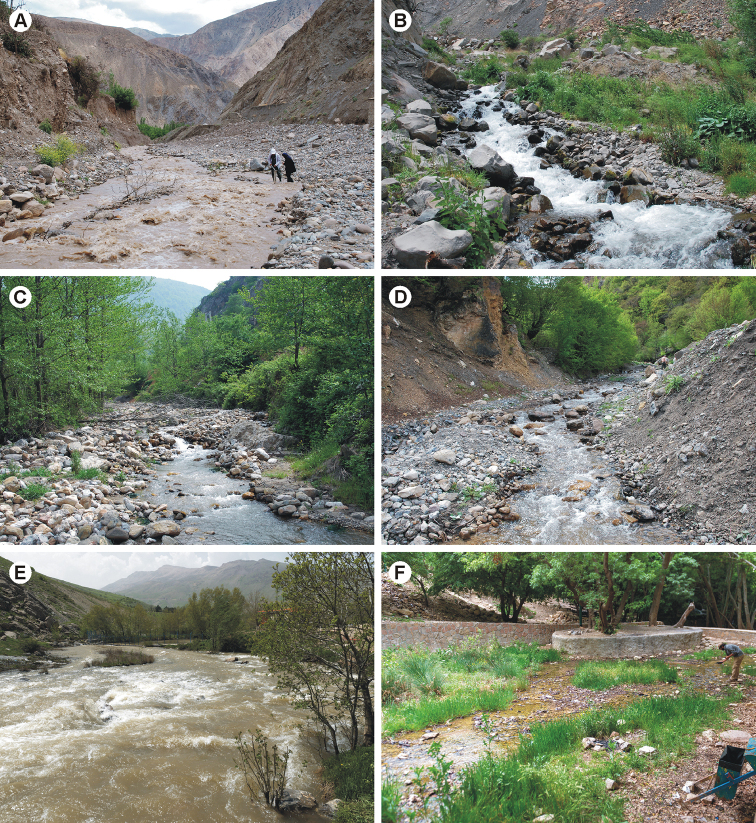
Photos of the localities of the new Epeorus (Caucasiron) species described herein: **A** unnamed brook near Panjab village – type locality of E. (C.) alborzicus sp. nov. **B** unnamed brook near Kahrud-e Bala village – locality of E. (C.) alborzicus sp. nov. **C** unnamed river near Shirinabad village – type locality of E. (C.) shargi sp. nov. **D** unnamed brook near Chah-e Ja village – locality of E. (C.) shargi sp. nov. **E** Gamasiab River near Varayeneh village – type locality of E. (C.) zagrosicus sp. nov. **F** Yasuj fall near Yasuj village – locality of E. (C.) zagrosicus sp. nov.

The presence of a medial hypodermal femur spot makes E. (C.) zagrosicus sp. nov. slightly similar to E. (C.) turcicus and E. (C.) alborzicus sp. nov. However, the presence of the characteristic pattern of abdominal sterna in E. (C.) alborzicus sp. nov. (Fig. 1B, G, K–M), and the absence of colouration pattern of abdominal sterna in E. (C.) turcicus reliably differentiate both species from E. (C.) zagrosicus sp. nov. Additionally, E. (C.) turcicus differs by the characteristic colour pattern of abdominal terga (Hrivniak et al. 2019: 61, fig. 1).

The other five species distributed in the Caucasus, namely E. (C.) sinitshenkovae, E. (C.) alpestris, E. (C.) bicolliculatus, E. (C.) longimaculatus, and E. (C.) soldani, do not share any important diagnostic characters with E. (C.) zagrosicus sp. nov. Nevertheless, E. (C.) sinitshenkovae and E. (C.) alpestris can be separated from E. (C.) zagrosicus sp. nov. by the absence of a medial hypodermal femur spot, overall colouration of the dorsal surface of femora (E. (C.) sinitshenkovae, Braasch and Zimmerman 1979: 106, fig. 10), and the different colouration of abdominal sterna (E. (C.) alpestris, Braasch 1979: 284, fig. 1d). Fine hair-like setae on the dorsal surface of abdominal terga clearly distinguish E. (C.) zagrosicus sp. nov. from E. (C.) bicolliculatus, E. (C.) longimaculatus, and E. (C.) soldani. All three species are characteristic by the presence of flattened setae on abdominal terga (Hrivniak et al. 2017: 359, figs 23–25).

### Results from molecular species delimitation

The GMYC model provided significantly better fit to COI gene tree than the null model expecting uniform coalescent branching rates across entire tree (likelihood ratio test = 3.671927e-06***). The GMYC estimated 15 species (CI=13–19) consisting of 14 ML clusters and one singleton (CI = 12–16). All three newly described species were confirmed, and the overall number of delimited GMYC species corresponded well to morphologically defined species within *Caucasiron* (Fig. 7C). Monophyly of all species clusters were highly supported (PP = 1).

The ABGD analysis of the COI distance matrix recognized 15 stable groups within initial partition. All groups corresponded well to morphologically defined species and were congruent with GMYC analysis. All three newly proposed species were recognized as distinct groups (Fig. 7C). The mean pairwise genetic K2P distances between all *Caucasiron* species, including newly described, ranged between 6.71% (E. (C.) caucasicus / E. (C.) iranicus) and 17.68% (E. (C.) alpestris / E. (C.) zagrosicus sp. nov.). Maximum intraspecific and minimum interspecific distances were observed in E. (C.) nigripilosus (4.12%; Iran/Cyprus) and E. (C.) caucasicus / E. (C.) iranicus (5.48%), respectively. Overall distribution of K2P pairwise genetic distances is figured on Fig. 7A. Mean intraspecific genetic distances for all new species relative to individual *Caucasiron* species are shown in Fig. 7B.

## Distribution of *Caucasiron* in Iran

Specimens of the genus *Epeorus* were found in 68 localities of all 254 localities investigated by us in 2016–2018 (Fig. 8) and in seven additional localities investigated by others (Braasch and Soldán 1979; Mousavi and Hakobyan 2017) (Table 1). Their occurrence was limited to streams with good water quality at altitudes between -4 and 2440 m a.s.l. (Table 1). They were neither found in polluted streams of agricultural and urban areas, nor in seasonally drying streams. Most of the species and records were found in the Alborz in northern Iran (Fig. 9). These mountains host five species of the subgenusCaucasiron and one species of the subgenusEpeorus (*E. zaitzevi*). Except for the newly described species, *E.* (*C.*) *nigripilosus* found in five localities in the Alborz is new for Iran (its genetic data from the Alborz were used in phylogeographical analyses in Hrivniak et al. 2020). It is a widely distributed species ranging from Cyprus and Turkey to Georgia, Russia and Iraq (Sinitshenkova, 1976; Braasch 1979; Al-Zubaidi et al. 1987; Salur et al. 2016; Gabelashvili et al. 2018; Hrivniak et al. 2020). The identification of E. (C.) nigripilosus was confirmed by both morphological characters and molecular delimitation. The specimen from the Alborz (coded as NI10I in Fig. 7C) clustered within the clade containing conspecific individuals from Russia and Turkey in the analysis of COI. It differed from these conspecifics in 2.8–3.6 % of K2P distance.

**Table 1.** List of records of the *Caucasiron* species found in Iran (three new species are not included). Abbreviations: RT – right tributary; LT – left tributary; JB – Jindřiška Bojková; TS – Tomáš Soldán; IN – Javid Imanpour Namin; SB – Samereh Bagheri; AHS – Arnold H. Staniczek; MP – Milan Pallmann; RJG – Roman J. Godunko; FN – Farshad Nejat; AA – Ashgar Abdoli; HV – H. Valikhani; PT – P. Taban. Number of specimens includes larvae.

Species	Province	Stream	Locality	Nearest settlement	Altitude	Latitude (N) / Longitude (E)	Sampling date	Collector/reference	Number of specimens
E. (C.) iranicus	Alborz	Karaj R., Shahrestanak branch	SE of Shahrestanak	Chavar Chalun	2220	35°57'45.8"N, 051°21'59.7"E	1.9.2016	AA, HV, PT	4
	Ardabil	unnamed brook	in Alvaresi (below Alvares ski area)	Sarein	2235	38°09'38.0"N, 047°56'21.0"E	17.5.2016	JB, TS, IN; Bojková et al. 2018	2
	Mazandaran	Koshk Sara R.	in Kosh Sara	Chalus	18	36°37'57.7"N, 051°28'04.4"E	25.9.2013	Mousavi and Hakobyan 2017	2
	Mazandaran	Firuz Abad R.	near Dasht Nazir	Marzan Abad	929	36°24'33.8"N, 051°24'42.5"E	11.9.2014	Mousavi and Hakobyan 2017	80
	Mazandaran	Haraz R.	in Gazanak	Gazanak	1590	35°54'08.3"N, 052°13'30.0"E	10.7.2013	Mousavi and Hakobyan 2017	8
	Mazandaran	Dalir R.	above Dalir	Marzan Abad	2126	36°19'23.2"N, 051°04'27.5"E	26.7.2014	Mousavi and Hakobyan 2017	174
	Mazandaran	Lasem R.	E of Polour	Polour	2180	35°50'04.1"N, 052°05'07.6"E	14.5.2017	AHS, MP, FN	1
	Mazandaran	RT of Sardab Rud	SW of Kelardasht	Kelardasht	2020	36°26'06.5"N, 051°03'52.6"E	8.3.2018	SB	85
	Mazandaran	LT of Sardab Rud	NW of Vandarbon	Kelardasht	2250	36°25'53.7"N, 051°01'59.1"E	9.3.2018	SB	63
	Mazandaran	Sardab Rud	S of Vandarbon	Kelardasht	2290	36°25'23.0"N, 051°02'12.4"E	10.3.2018	SB	5
	Tehran	Darban valley (type locality)	N of Tehran	Tehran	2100	35°50'24.0"N, 051°25'19.9"E	18.7.1970	Braasch and Soldán 1979	14
	Tehran	Lalan R.	above Zayegan	Fasham	2290	35°58'39.2"N, 051°34'56.5"E	8.5.2017	AHS, MP, RJG, FN	158
	Tehran	Lalan R.	in Lalan	Lalan	2440	35°59'50.3"N, 051°34'51.0"E	8.5.2017	AHS, MP, RJG, FN	87
	Tehran	Ahar R.	near Igol	Fasham	2020	35°55'11.2"N, 051°28'51.3"E	8.5.2017	AHS, MP, RJG, FN	1
	Tehran	Shahrestanak R.	NW of Shahrestanak	Asara	2100	35°59'01.2"N, 051°19'09.6"E	10.5.2017	AHS, MP, FN	1
E. (C.) nigripilosus	Alborz	Kordan R.	N of Kordan	Kordan	1430	35°57'15.6"N, 050°50'25.3"E	10.5.2017	AHS, MP, FN	4
	Mazandaran	RT of Dohezar R.	N of Holu Kaleh	Tonkaboon	880	36°37'37.5"N, 050°44'30.2"E	16.6.2018	SB	1
	Mazandaran	Dohezar R.	SW of Parde Sar	Tonkaboon	450	36°40'07.0"N, 050°49'20.0"E	16.6.2018	SB	1
	Tehran	Ahar R.	near Igol	Fasham	2020	35°55'11.2"N, 051°28'51.3"E	8.5.2017	AHS, MP, RJG, FN	5
	Tehran	Shahrestanak R.	NW of Shahrestanak	Asara	2100	35°59'01.2"N, 051°19'09.6"E	10.5.2017	AHS, MP, FN	1
E. (C.) cf. znojkoi	Alborz	Kordan R.	N of Kordan	Kordan	1430	35°57'15.6"N, 050°50'25.3"E	10.5.2017	AHS, MP, FN	4
	Gilan	RT of Khara Rud	S of Paein Khara Rud (S of Pashaki)	Sangar	210	37°02'29.0"N, 049°47'52.0"E	12.5.2016	JB, TS, IN	71
	Gilan	left fork of Khara Rud	in Madarsara (S of Pashaki)	Sangar	105	37°04'12.0"N, 049°46'36.0"E	12.5.2016	JB, TS, IN	27
	Gilan	right fork of Khara Rud	in Golestansara (S of Pashaki)	Sangar	201	37°02'20.0"N, 049°47'27.0"E	12.5.2016	JB, TS, IN	2
	Gilan	Zilaki River (RT of Sefid Rud)	in Mush Bijar (E of Shahr-e Bijar)	Shahr-e Bijar	120	37°00'28.0"N, 049°40'24.0"E	13.5.2016	JB, TS, IN	1
	Gilan	Sefidab (RT of Siah Rud)	in Divarsh (NE of Shirkuh)	Tutkabon	280	36°53'59.0"N, 049°35'06.0"E	13.5.2016	JB, TS, IN	149
	Gilan	Sangdeh (LT of Shafa Rud)	W of Punel	Punel	240	37°31'47.0"N, 049°00'52.0"E	15.5.2016	JB, TS, IN	10
	Gilan	Shafa Rud	W of Punel	Punel	240	37°31'47.0"N, 049°00'52.0"E	15.5.2016	JB, TS, IN	6
	Gilan	LT of Shafa Rud	NW of Sangdeh	Sangdeh	1345	37°31'46.0"N, 048°45'19.0"E	15.5.2016	JB, TS, IN	50
	Gilan	Shakhzar R.	NE of Fuman	Fuman	6	37°14'13.0"N, 049°20'43.0"E	15.5.2016	JB, TS, IN	1
	Gilan	LT of Bala Rud	S of Siahkal	Siahkal	490	37°00'31.0"N, 049°51'51.0"E	16.5.2016	JB, TS, IN	16
	Gilan	Lunak waterfalls	S of Siahkal	Siahkal	510	37°00'31.0"N, 049°51'49.0"E	16.5.2016	JB, TS, IN	5
	Gilan	Shamrud (RT of Sefid Rud)	S of Tushi (S of Siahkal)	Siahkal	315	37°03'00.0"N, 049°53'54.0"E	16.5.2016	JB, TS, IN	37
	Gilan	Chelavand R.	W of Chelvand	Lavandvil	-4	38°17'20.0"N, 048°51'35.0"E	19.5.2016	JB, TS, IN	2
	Gilan	unnamed brook	N of Chaldasht	Amlash	1255	36°59'33.0"N, 050°05'19.0"E	21.5.2016	JB, TS, IN	3
	Gilan	RT of Shalman Rud	in Bolurdekan	Amlash	345	37°01'09.0"N, 050°03'51.0"E	21.5.2016	JB, TS, IN	2
	Gilan	LT of Ghale Rudkhan	NE of Masuleh	Fuman	885	37°09'47.0"N, 049°00'17.0"E	22.5.2016	JB, TS, IN	66
	Gilan	RT of Ghale Rudkhan	NE of Masuleh	Fuman	705	37°09'42.0"N, 049°01'17.0"E	22.5.2016	JB, TS, IN	6
	Gilan	Ghale Rudkhan R.	E of Masuleh	Fuman	370	37°10'02.0"N, 049°05'03.0"E	22.5.2016	JB, TS, IN	4
	Golestan	unnamed river	in Shirinabad	Aliabad-e Katul	740	36°48'01.0"N, 055°01'05.0"E	27.4.2018	JB, TS, IN	50
	Golestan	Shirabad waterfalls	above Shirabad	Shirabad	140	36°57'33.0"N, 055°01'57.0"E	28.4.2018	JB, TS, IN	48
	Golestan	RT of Madarsu R.	E of Tangrah	Tangrah	495	37°23'27.0"N, 055°48'51.0"E	30.4.2018	JB, TS, IN	1
E. (C.) cf. znojkoi	Mazandaran	Shirinrud	S of Part Kola	Farim	770	36°09'02.5"N, 053°20'58.1"E	11.5.2017	AHS, MP, FN	203
	Mazandaran	trib. Kashpel R.	SW of Chamestan	Chamestan	400	36°25'31.4"N, 052°03'38.4"E	13.5.2017	AHS, MP, FN	17
	Mazandaran	Chelav R.	N of Pasha Kola	Pasha Kola	820	36°12'24.7"N, 052°25'51.9"E	14.5.2017	AHS, MP, FN	63
	Mazandaran	Chelav R.	NW of Pasha Kola	Pasha Kola	570	36°13'28.9"N, 052°23'35.4"E	14.5.2017	AHS, MP, FN	2
	Mazandaran	Baladeh R.	W of Razan	Razan	1360	36°11'39.6"N, 052°08'34.6"E	14.5.2017	AHS, MP, FN	9
	Mazandaran	Chai Bagh R.	E of Andar Koli	Ghaem Shahr	200	36°20'30.0"N, 052°54'03.0"E	8.5.2018	JB, TS, IN, SB	27
	Mazandaran	RT of Haraz R.	NW of Pasha Kola	Amol	570	36°13'27.0"N, 052°23'36.0"E	9.5.2018	JB, TS, IN, SB	5
	Mazandaran	LT of Haraz R.	in Panjab	Amol	955	36°05'52.0"N, 052°15'15.0"E	9.5.2018	JB, TS, IN, SB	1
	Mazandaran	unnamed brook	above Darab Kola	Neka	135	36°33'10.0"N, 053°15'32.0"E	10.5.2018	JB, TS, SB	2
	Mazandaran	unnamed brook	in Momey Khal	Ghaem Shahr	760	36°04'24.0"N, 052°58'19.0"E	11.5.2018	JB, TS, SB	1
	Mazandaran	Palang Darreh R.	SE of Shirgah	Shirgah	320	36°16'31.0"N, 052°56'54.0"E	11.5.2018	JB, TS, SB	2
	Mazandaran	LT of Palang Darreh R.	SE of Shirgah	Shirgah	345	36°16'30.0"N, 052°56'51.0"E	11.5.2018	JB, TS, SB	1
	Mazandaran	RT of Sardab Rud	SW of Kelardasht	Kelardasht	2020	36°26'06.5"N, 051°03'52.6"E	9.3.2018	SB	9
	Mazandaran	Sardab Rud	S of Vandarbon	Kelardasht	2290	36°25'23.0"N, 051°02'12.4"E	16.6.2018	SB	57
	Mazandaran	RT of Dohezar R.	N Holu Kaleh	Tonkabon	880	36°37'37.5"N, 050°44'30.2"E	16.6.2018	SB	83
	Mazandaran	Dohezar R.	SW Parde Sar	Tonkabon	450	36°40'07.0"N, 050°49'20.0"E	16.6.2018	SB	46
	Mazandaran	RT of Sehezar R.	S Parde Sar	Tonkabon	570	36°38'41.5"N, 050°50'11.1"E	16.6.2018	SB	20
	Mazandaran	Sehezar R.	S Parde Sar	Tonkabon	540	36°39'01.0"N, 050°50'00.0"E	24.8.2018	SB	21
	Mazandaran	Lavij Rud	SE of Kiakola	Noor	820	36°21'33.1"N, 052°03'11.0"E	24.8.2018	SB	8
	Mazandaran	Vaz Rud	E of Vaz Oliya	Noor	1140	36°19'08.0"N, 052°08'24.1"E	15.6.2018	SB	33
	Mazandaran	Safarud	SW of Ramsar	Ramsar	490	36°52'55.8"N, 050°33'56.1"E	15.6.2018	SB	1
	Mazandaran	LT of Safarud	SW of Ramsar	Ramsar	610	36°53'24.4"N, 050°33'56.1"E	15.6.2018	SB	2
	Mazandaran	LT of Safarud	SW of Ramsar	Ramsar	330	36°54'06.1"N, 050°35'12.1"E	15.6.2018	SB	11
	Mazandaran	Chalak Rud	SW of Galeshmahalleh	Ramsar	100	36°49'13.0"N, 050°43'23.8"E	15.6.2018	SB	3
	Mazandaran	LT of Chalak Rud	NW of Talesh Sara	Ramsar	180	36°50'46.9"N, 050°40'25.9"E	8.3.2018	SB	8
	Tehran	Ahar R.	near Igol	Fasham	2020	35°55'11.2"N, 051°28'51.3"E	8.5.2017	AHS, MP, RJG, FN	4

The most common *Caucasiron* species in the Alborz is E. (C.) cf. znojkoi distributed from the Talysh Mts. in the west to the Golestan NP in the east (Fig. 9). However, our study dealing with the molecular diversity of *Caucasiron* species in the Caucasus and adjacent regions (Hrivniak et al. 2020) indicated that E. (C.) znojkoi might represent a complex of cryptic species (only a subset of sequences included in the present study). The lineage *Caucasiron* sp. 4 (see Hrivniak et al. 2020) occurring in Iran (here called E. (C.) cf. znojkoi) differed from the Central Caucasian lineage. The delimitation of species within E. (C.) znojkoi s. l. requires further study. Nevertheless, the morphotype of E. (C.) cf. znojkoi has a wide geographical and ecological range, occurring at altitudes from -4 to 2290 m a.s.l. in northern Iran (Table 1). It was often found in shallow warm streams with good water quality flowing in humid broadleaved forests in the Caspian Sea lowland; approximately half of its localities was below 350 m a.s.l. At higher altitude, it can co-occur with E. (C.) alborzicus sp. nov., E. (C.) shargi sp. nov., E. (C.) nigripilosus, and E. (C.) iranicus (Fig. 9).

Three *Caucasiron* species, E. (C.) iranicus, E. (C.) alborzicus sp. nov., and E. (C.) shargi sp. nov., were described from the Alborz and are so far only known from there. E. (C.) iranicus is reliably reported from 12 localities, eight of them above 2000 m a.s.l. These include the Sabalan Mt. slopes in the western Alborz and the central Alborz, where it can co-occur with E. (C.) alborzicus sp. nov. (Fig. 9). It was found only in very cold streams fed by glaciers and melting snow from the highest mountains, with very rapid flow and strongly turbulent riffle sections. Four records of E. (C.) iranicus published by Mousavi and Hakobyan (2017) should be revised, because they included a wide range of altitude (20–2120 m a.s.l.) and were very close to our records of E. (C.) alborzicus sp. nov. and E. (C.) cf. znojkoi. The two new species from the Alborz seem to differ in habitat requirements. Epeorus (C.) alborzicus sp. nov. was only found in higher altitudes. These were all treeless localities in montane valleys with harsh climatic conditions, whereas E. (C.) shargi sp. nov. was found well below in submontane streams that were flowing in forests. The latter species was recorded only in the eastern Alborz near Gorgan (Fig. 9).

Other streams investigated in Iranian mountain ranges were dominated by Baetidae, and Heptageniidae were generally only scattered there. Larvae of E. (C.) zagrosicus sp. nov. and E. (E.) zaitzevi were found only in five and seven localities respectively, relatively distant to each other in the Zagros (Fig. 8). However, most of the streams explored in the Zagros were polluted or seasonally drying out due to the water storage in dams and water abstraction for irrigation of surrounding fields. Moreover, streams at higher altitude with presumably better water quality were almost inaccessible for us in April and May during our field trips. As E. (C.) zagrosicus sp. nov. was mostly found in natural streams in high-mountain valleys only with sparse villages, we expect that its distribution is limited to clear and cold mountain streams. However, a more detailed investigation of mayflies in high-mountain streams in Iran is needed.

## Supplementary Material

XML Treatment for Epeorus (Caucasiron) alborzicus

XML Treatment for Epeorus (Caucasiron) shargi

XML Treatment for Epeorus (Caucasiron) zagrosicus
